# Time-Course Proteomic Analysis of *Pseudomonas putida* KT2440 during Mcl-Polyhydroxyalkanoate Synthesis under Nitrogen Deficiency

**DOI:** 10.3390/polym11050748

**Published:** 2019-04-26

**Authors:** Justyna Możejko-Ciesielska, Agnieszka Mostek

**Affiliations:** 1 Department of Microbiology and Mycology, Faculty of Biology and Biotechnology, University of Warmia and Mazury in Olsztyn, Oczapowskiego 1A, 10719 Olsztyn, Poland; 2 Department of Gamete and Embryo Biology, Institute of Animal Reproduction and Food Research, Polish Academy of Sciences in Olsztyn, Tuwima 10, 10748 Olsztyn, Poland; a.mostek@pan.olsztyn.pl

**Keywords:** polyhydroxyalkanoates, mcl-PHA, *Pseudomonas putida* KT2440, proteomics

## Abstract

Medium-chain-length polyhydroxyalkanoates (mcl-PHAs) have gained great attention as a new green alternative to petrochemical-derived polymers. Due to their outstanding material properties they can be used in a wide range of applications. *Pseudomonas putida* KT2440 is a metabolically versatile producer of mcl-polyhydroxyalkanoates. Although the metabolism of polyhydroxyalkanoate synthesis by this bacterium has been extensively studied, the comparative proteome analysis from three growth stages of *Pseudomonas putida* KT2440 cultured with oleic acid during mcl-PHA synthesis has not yet been reported. Therefore; the aim of the study was to compare the proteome of *Pseudomonas putida* KT2440 at different time points of its cultivation using the 2D difference gel electrophoresis (2D-DIGE) technique. The analyses showed that low levels of a nitrogen source were beneficial for mcl-PHA synthesis. Proteomic analysis revealed that the proteins associated with carbon metabolism were affected by nitrogen starvation and mcl-PHA synthesis. Furthermore, the induction of proteins involved in nitrogen metabolism, ribosome synthesis, and transport was observed, which may be the cellular response to stress related to nitrogen deficiency and mcl-PHA content in bacterial cells. To sum up; this study enabled the investigators to acquire a better knowledge of the molecular mechanisms underlying the induction of polyhydroxyalkanoate synthesis and accumulation in *Pseudomonas putida* KT2440 that could lead to improved strategies for PHAs in industrial production.

## 1. Introduction

Synthetic plastics are considered one of the most problematic issues, and this has led to a growing interest in developing alternative materials that are environmental friendly. There is a need for technologies that will enable the economical production of biodegradable polymers. Among the biodegradable polymers, the various types of biopolymers polyhydroxyalkanoates (PHAs) are especially attractive due to their outstanding material properties, including not only biodegradability but also bio-renewability, thermal plasticity, and biocompatibility. Moreover, they have great potential as a new functional material in biomedicine, agricultural, and industrial applications [1,2]. PHAs are natural polyesters synthesized by a variety of bacteria as carbon and energy reserves, thus ensuring their survival during nutritional stress [3]. Culture stress is becoming an efficient strategy for the biosynthesis of polyhydroxyalkanoates by bacteria. It has been previously reported that nutrient limitation stimulates rapid PHA synthesis [4].

Generally, polyhydroxyalkanoates are classified into two groups according to the number of carbon atoms in their monomeric units: short-chain-length (scl-PHAs; 3–5 carbon atoms) and medium-chain-length (mcl-PHAs; more than 6 carbon atoms). The type of this biopolyester depends on the carbon source used for bacterial growth and on the host microorganism that is able to synthesize and accumulate PHAs. Scl-PHAs have been produced on a commercial scale and studied in depth. Nevertheless, due to high crystallinity and poor elastic properties their use in a wide range of applications is limited. Therefore, mcl-PHAs having more favorable properties, have gained much interest in research. They are mainly synthesized by bacteria from the genus *Pseudomonas* possessing the genetic machinery that determines their ability to accumulate these biopolymers from a variety of carbon sources [5]. Among these bacteria, *Pseudomonas putida* KT2440 is considered as a model candidate to investigate PHAs synthesis on a molecular level, since the sequencing and publication of its complete genome [6].

Although PHAs have attracted much attention in recent years, the molecular regulation of their synthesis is still unclear. Many biofermentations towards biotechnological mcl-PHA synthesis were conducted using fatty acids as the only substrate in the medium [7,8]. The regulatory mechanisms that drive PHA synthesis in *Pseudomonas* species were previously studied at the transcriptomic level [9,10,11]. The proteome of *Pseudomonas putida* KT2442 [12] and *Pseudomonas putida* LS46 [13] during mcl-PHA synthesis have been reported for sodium decanoate and waste glycerol or waste fatty acids, respectively. The comparative proteome analysis from three growth and PHA synthesis stages of *Pseudomonas putida* KT2440 cultured with oleic acid during mcl-PHA synthesis has not yet been reported.

Therefore, to provide new insights into the mechanism of PHAs accumulation, we have studied *Pseudomonas putida* KT2440 proteome during nitrogen starvation using a novel time-course 2D difference gel electrophoresis (2D-DIGE) proteomic technique. This study has been applied to understand the potential genetic targets associated with biopolyester synthesis inside bacterial cells.

## 2. Materials and Methods

### 2.1. Microorganisms and Culture Media

*Pseudomonas putida* KT2440 (ATCC^®^ 47054^TM^) from long-term storage tubes (in nutrient broth containing 30% glycerol) were grown in lysogeny broth (10 g/L tryptone, 5 g/L yeast extract, 10 g/L NaCl) at 30 °C with 220 rpm shaking for 16 h before inoculation into a bioreactor. The mineral medium for PHA synthesis under limited-conditions contained the following components (per liter): 3.5 g Na_2_HPO_4_·12H_2_O, 7.0 g KH_2_PO_4_, 1 g (NH_4_)SO_4_. Whereas non-limiting medium consisted of (per liter): 3.5 g Na_2_HPO_4_·12H_2_O, 7.0 g KH_2_PO_4_, 10 g (NH_4_)SO_4_. Each medium was supplemented with 1 g/L MgSO_4_·7H_2_O and 2.5 mL/L of trace element solution. Each liter of trace element solution consisted of (per liter): 20 g FeCl_3_·6H_2_O, 10 g CaCl_2_·H_2_O, 0.03 g CuSO_4_·5H_2_O, 0.05 g MnCl_2_·4H_2_O, 0.1 g ZnSO_4_·7H_2_O dissolved in 0.5 N HCL. All medium components were dissolved in water and then autoclaved at 121°C. MgSO_4_·7H_2_O was sterilized separately. Oleic acid was used as the only carbon source in the production medium. The pulsed feeding cultivations were performed with the initial concentration of 5 mL/L of the oleic acid. After 17 h and 24 h of the biofermentations, additional substrate ratios were added into the PHA synthesis medium in the amount of 2.5 mL/L. The total concentration of the carbon source was 10 mL/L.

### 2.2. Fermentation Conditions

The precultures of *Pseudomonas putida* KT2440 were used to inoculate a 7.0-L bioreactor (Biostat A, Sartorius, Germany) so that the initial OD_600_ was 0.1. The inoculation size was 10%. All cultivations were carried out at 30 °C. The temperature was maintained by a thermostatic jacket. The pH of each culture was maintained at 7.0 through the modulated addition of 1 N NaOH and 1 N HCl. The dissolved oxygen was monitored during the entire cycle with an O_2_ electrode (InPro 6800, Mettler Toledo GmbH, Greifensee, Switzerland). The agitation rate from 300 rpm to 1000 rpm was adjusted automatically to maintain 50% air saturation. The total fermentation time was 48 h. An antifoam solution (Sigma Aldrich, St. Louis, MO, USA) was used in response to the antifoam controller.

### 2.3. Analytical Methods

During the fermentations, samples of the culture broth were taken at 8, 17, 24, 32, 41, and 48 h for measurements of cell dry weight, PHAs concentration, ammonium concentration, and for determination of mcl-PHAs composition and monomers concentrations. To measure cell dry weight (CDW), 100 mL culture broth were centrifuged at 11,200× *g* for 10 min and washed with hexane and distilled water to remove hydrophobic oleic acid. The bacterial cells were then lyophilized by a Lyovac GT2 System (SRK Systemtechnik GmbH) for 24 h. After the lyophilization process the collected cells were weighed. Ammonium concentration was determined spectrophotometrically using a Hach Lange DR 2800 spectrophotometer (Hach Lange, Düsseldorf, Germany) and the LCK303 kit according to the manufacturer’s instructions.

### 2.4. Mcl-PHAs Extraction and Analysis

Quantitative and qualitative analysis of the extracted PHAs was conducted. Biopolyesters were isolated from lyophilized cells by vigorous shaking with chloroform for 3 h at 50 °C. Then, the mixture was filtered through Whatman no. 1 filter paper. The crude biopolymer was allowed to evaporate at room temperature. The PHAs content was calculated as the percentage of the ratio of PHAs concentration to cell dry weight. 

The monomeric composition of the purified mcl-PHAs was determined using a methanolysis protocol as described previously [14]. The concentrations of methyl esters were estimated by gas chromatography (GC) using a capillary column Varian VF-5 ms with a film thickness of 0.25 μm (Varian, Lake Forest, CA, USA). Firstly, the extracted polymers were suspended in 2 mL of acidified methanol and an equal volume of chloroform. The esterification process was performed in the oven for 20 h at 100 °C. Pure standards of methyl 3-hydroxyhexanoate, -octanoate, -nonanoate, -decanoate, -undecanoate, -dodecanoate, -tetradecanoate, -hexadecanoate were purchased from Larodan Fine Chemicals (Solna, Sweden) to generate calibration curves for the methanolysis assay. All samples were analyzed in triplicates.

### 2.5. Protein Extraction and Labeling with Fluorescent Dyes 

Prior to proteomic analysis, the fresh bacterial cells were harvested from the cultures at 8 h, 24 h, and 48 h, and centrifuged at 10,000× *g* at 4 °C for 5 min. The obtained pellets were resuspended in 50 μL of lysis buffer containing 7 M urea, 2 M thiourea, 2% 3-[(3-cholamidopropyl)-dimethylammonio]-1-propanesulfonate (CHAPS), 2% [v/v] immobilised pH gradient (IPG) buffer 3–10 NL, 50 mM dithiothreitol (DTT), and 0.5% (v/v) protease inhibitor cocktail (Sigma-Aldrich, St. Louis, MO, USA). Aliquots containing approximately 800 μg of bacterial proteins were processed using a Clean-Up Kit (GE Healthcare, Uppsala, Sweden) according to the manufacturer’s protocol. The protein concentration prior to and after the cleaning procedure was measured by a Coomassie (Bradford) Protein Assay Kit (Thermo Scientific, Waltham, MA, USA).

The purified protein pellets were resuspended in DIGE Labelling Buffer (7 M urea, 2 M thiourea, 4% w/v CHAPS and 30 mM Tris, pH 8) to a protein concentration of 5–10 mg/mL. Aliquots containing 50 μg of protein from each sample were labeled with 400 pmol of CyDye DIGE Fluor minimal dyes (GE Healthcare, Uppsala, Sweden) reconstituted in fresh 99.8% anhydrous DMF. Cy2 was used to label the internal standard, while Cy3 and Cy5 were used to label individual samples. An internal standard was created by mixing equal amounts of protein from all samples. The labeling reaction was performed in the dark on ice for 30 min. Samples labeled with Cy3 (50 µg) were mixed with samples labeled with Cy5 (50 µg) and 50 µg of Cy2-labeled internal standard, and then rehydration solution was added (7 M urea, 2 M thiourea, 2% CHAPS, DDT, 2% pharmalyte pH 3–10, and 130 mM DTT) to a final volume of 450 µL.

### 2.6. D-DIGE 

Protein samples were loaded onto 24-cm Immobiline DryStrips, with a 3–10 nonlinear gradient pH range (GE Healthcare, Uppsala, Sweden), and rehydrated for 12 h. In the first dimension of electrophoresis, the proteins were separated according to their isoelectric point using an Ettan IPGphor apparatus (GE Healthcare, Uppsala, Sweden) at 20 °C with current limited to 50 µA per strip and the following voltage program: 500 V over 2 h, a linear gradient to 1000 V over 1 h and a linear gradient to 10,000 V over 3 h, then at 10,000 V constant for 4 h. Subsequently, the strips were equilibrated in SDS equilibration buffer (6 M urea, 75 mM Tris-HCl, pH 8.8, 29.3% glycerol, 2% sodium dodecyl sulfate, 0.002% bromophenol blue) containing 65 mM DDT for 15 min, and then in SDS equilibration buffer containing 135 mM iodoacetamide for 15 min. The equilibrated strips were then transferred to precast DIGE gels Ettan DALT Gel 12.5 (25.5 × 19.6 cm, 1 mm thickness, GE Healthcare, Uppsala, Sweden) and sealed with 0.5% agarose. A second dimension of electrophoresis was then performed at 1.5 W/gel in an Ettan Dalt-Six apparatus (GE Healthcare, Uppsala, Sweden) for 16 h. The gels were scanned using a Typhoon 9500 FLA scanner (GE Healthcare, Uppsala, Sweden) and the obtained gel images were subjected to a statistical analysis.

### 2.7. Image Acquisition and Analysis

The gel images depicting protein maps were analyzed using the SameSpots software (Totallab, Newcastle, UK) to identify differences in the fluorescence intensities of the protein spots corresponding to different time-points of the cultivation. To provide statistical data on the differential protein expression, the obtained protein spot maps corresponding to biological replicates were analyzed using one-way ANOVA. Spots that exhibited at least 2-fold change of abundance and a *p*-value < 0.05 were considered as differentially regulated.

To show differences between samples principal component analysis (PCA) and hierarchical clustering were performed using a web-based ClustalVis software (Institute of Computer Science, Tartu, Estonia). The heatmaps were conducted to group samples with similar expression patterns into clusters. PCA creates and coordinates those points using the total log standardized abundance of spots on a certain gel. This approach allowed the investigators to summarize the variation in the data from protein spots in the form of principal components, where the first component explains the largest proportion of the variance.

### 2.8. Protein Digestion and MALDI TOF/TOF Protein Identification

DIGE gels were restained using Coomassie Brilliant Blue G-250 (Bio-Rad, Hercules, CA, USA) in order to properly pick the differentially expressed proteins. Spots that exhibited significant abundance changes were manually cut out from the gels, placed in Eppendorf tubes and washed with 100 μL of 50 mM ammonium bicarbonate. The solution was discarded, and the spots were washed again with 50 μL of 50 mM ammonium bicarbonate in 50% acetone. After the solution was discarded and the spots were dried, 5–10 μL (depending on spot size) of 0.2 μg/μL modified sequencing grade trypsin (Promega, Madison, WI, USA) in 20 mM ammonium bicarbonate was added, and spots were incubated for 12 h at 37 °C. After digestion, the spots were placed in 100 μL of 0.1% trifluoroacetic acid (TFA) and desalted with Zip-Tip C-18 pipette tips (Millipore, Billerica, MA, USA). Each Zip-Tip was first washed with 100% acetonitrile (ACN), then equilibrated with 50% ACN in 0.1% TFA and 0.1% TFA in water. Then, the peptides were loaded onto the Zip-tip and eluted with 2 μL of 50% ACN in 0.1% TFA. The eluted samples were mixed with 2 μL of the matrix solution (5 mg α-cyano-4-hydroxycinnamic acid (Bruker Daltonics, Bremen, Germany) in 1 mL of 50% ACN in 0% TFA). The mixture was spotted onto the MALDI target plate (MT 34 Target Plate Ground Steel (Bruker Daltonics, Bremen, Germany) and left to dry.

Peptide samples were analyzed using matrix-assisted laser desorption/ionization time of-flight/time-of-flight spectrometry (MALDI-TOF/TOF) with Autoflex mass spectrometer (Bruker Daltonics, Germany). The MS spectra, together with the MS/MS spectra, were searched using the Mascot Server (Matrix Science, London, UK) of the National Centre for Biotechnology Information database. Search parameters were as follows: trypsin cleavage, monoisotopic masses MH+, two missed cleavage sites allowed, carbamidomethylation of cysteine as the fixed modification, oxidation of methionine as the variable modification, fragment ion mass tolerance of 0.5 Da and parent ion mass tolerance of 50 ppm. Matches considered statistically significant (*p* < 0.05) by Mascot with at least two correctly identified parent ions were regarded as correct hits.

## 3. Results and Discussion

### 3.1. Fed-Batch Fermentation

**Figure 1:**
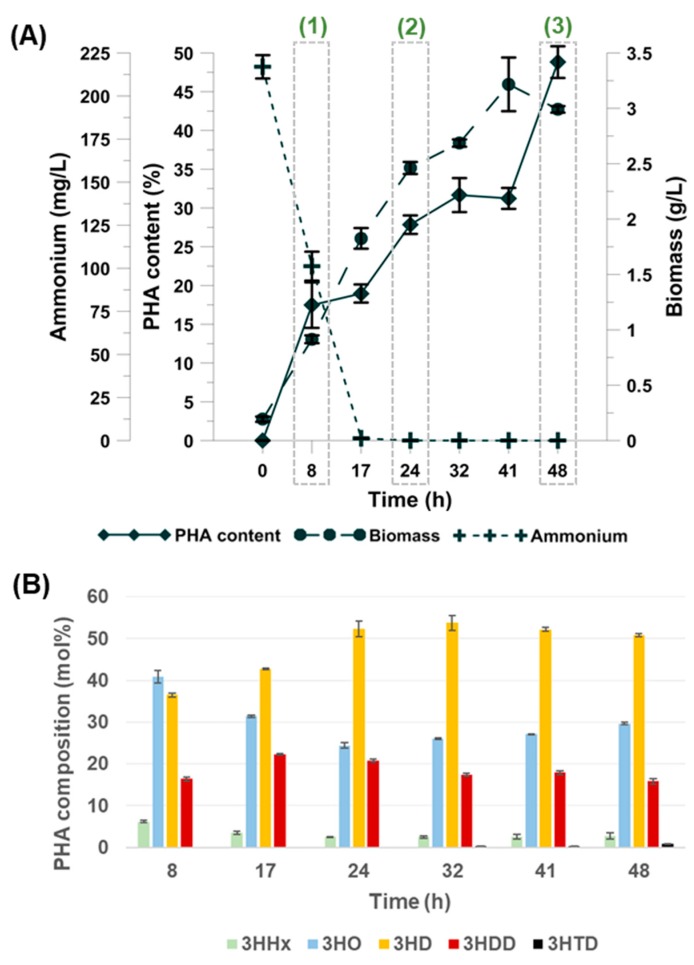
(**A**) Medium-chain-length polyhydroxyalkanoates (mcl-PHAs) content, biomass, and ammonium concentration during fed-batch fermentations of *Pseudomonas putida* KT2440. (1), (2), and (3) indicate bacterial samples collected for proteomic analysis at 8 h, 24 h, and 48 h of the cultivation, respectively. (**B**) Monomeric composition of mcl-PHAs produced by *Pseudomonas putida* KT2440 in bioreactor experiments. Mean values are calculated from triplicate measurements. 3HHx, 3-hydroxyhexanoic acid; 3HO, 3-hydroxyoctanoic acid; 3HD, 3-hydroxydecanoic acid; 3HDD, 3-hydroxydodecanoic acid; 3HTD, 3-hydroxy-tetradecanoic acid.

According to the literature, a carbon source provided in excess could stimulate intracellular accumulation of biopolyesters under limiting conditions of other nutrients [15]. In this study, to evaluate the cellular responses during polyhydroxyalkanoates synthesis, *Pseudomonas putida* KT2440 was grown in nitrogen-limited mineral salt medium containing oleic acid as a carbon source. As shown in Figure 1A oleic acid could support good cell growth and mcl-PHAs accumulation. Biomass concentration increased rapidly up to 41 h, and then slowed down reaching 3 g/L at the end of the bioprocess. Ammonium was used up at about 17 h. Low levels of a nitrogen source were beneficial for mcl-PHAs synthesis. After nitrogen depletion from the growth medium, *Pseudomonas putida* KT2440 continuously accumulated mcl-PHAs up to 48 h of the fermentation. A maximum mcl-PHAs content of 48.8% of CDW was reached at the end of the cultivation. Lower PHAs yield (29.7% of CDW) was observed during growth of the same strain on glycerol under nitrogen limitation [16]. Furthermore, it was reported that *P. putida* KT2442 cultivated on sodium decanoate under strict nitrogen-limited conditions was able to synthesize up to 81% of PHAs [12]. However, Follonier et al. [17] demonstrated that an effect of a pleiotropic mutation in KT2442 strain could reach deep into its physiological regulation and as a consequence converts *P. putida* KT2440 and KT2442 into two different mcl-PHAs producers.

*Pseudomonas putida* KT2440 was also cultivated in a bioreactor under non-limiting conditions. The aim of this cultivation was to take a sample that served as a control for proteomic analysis. Our results revealed that during optimal growth conditions *P. putida* was capable of synthesizing from 2.5% to 5.6% of PHAs. The trace amounts of PHAs could have arisen from the contamination with cell membrane components and was treated as not to be actual biopolymer [18]. The data suggested that the analyzed strain was not able to accumulate PHAs without nitrogen starvation. Therefore, we decided to harvest a sample at the beginning of the bioprocess (8 h) when ammonium was at the level of 1800 mg/L and no PHAs in bacterial cells were detected. 

### 3.2. PHAs Composition 

Biopolymers synthesized by *Pseudomonas putida* KT2440 were isolated and purified to determine their monomeric composition by GC analysis. The data in the literature suggested that PHA producers growing on fatty acids consisting of 13- to 18-carbon synthesize monomers containing 8- to 10-carbon units [19]. As shown in Figure 1B, the monomers composition and their distribution varied with the progress of the fermentation process. The obtained results revealed that mcl-PHAs produced by *P. putida* KT2440 were found to consist mainly of 3-hydroxyoctanoate (3-HO) and 3-hydroxydecanoate (3-HD). The analyzed strain showed also a tendency to accumulate lesser amounts of 3-hydroxydodecanoate (3-HDD) and 3-hydroxyhexanoate (3-HHx). Furthermore, the data confirmed that nitrogen stress induced some changes in the mcl-PHAs composition and distribution profile. It was observed that after ammonia depletion, a molar fraction of 3-HD increased from 36.5 mol.% at 8 h to 50 mol.% at the end of the cultivation. Nutrient-limited regimes seem to have an influence on C10 concentration in PHAs extracted from a chemostat during the growth of *P. putida* KT2440 on decanoate [12]. Furthermore, our results suggest that nitrogen starvation could induce the synthesis of 3-hydroxytetradecanoate (3-HTD). This fraction was produced from 32 h to 48 h, albeit in trace amounts from 0.4 to 0.9 mol.%, respectively.

### 3.3. Proteome Changes during PHAs Synthesis under Nitrogen Starvation

Considering the results obtained during the fermentation process (Figure 1A) and in order to investigate the differentially expressed proteins during mcl-PHAs synthesis triggered with nitrogen depletion, the bacterial cells were taken from the bioreactor at 8 h (low concentrations of ammonium and mcl-PHAs, growth phase), 24 h (7 h after nitrogen depletion, fast mcl-PHAs synthesis, exponential phase), and 48 h (the highest mcl-PHAs content, stationary phase). The bacterial sample harvested at 8 h of the non-limiting cultivation was treated as a control. This time point is one where PHAs were not detected and the nitrogen source was provided at the optimal level. To examine how proteome profile changed in response to biopolymers synthesis and accumulation under nutrient stress, the protein expression profiles of the samples collected at 8, 24, and 48 h were compared to this at 8 h of the non-limiting growth conditions.

**Figure 2:**
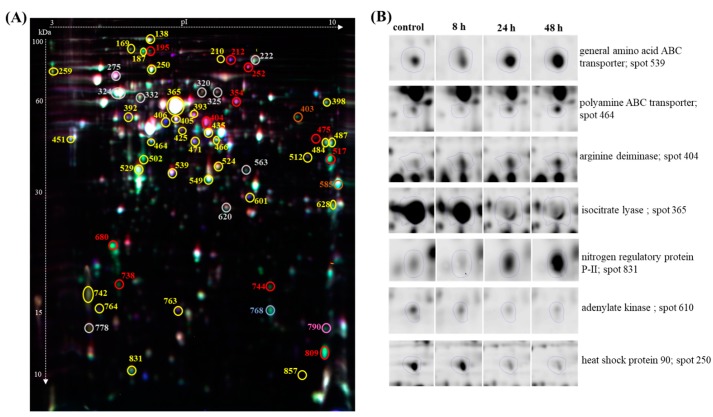
Comparative 2D difference gel electrophoresis (2D-DIGE) analysis of cellular proteins of *Pseudomonas putida* KT2440. Numbers indicate spots that significantly differ at 8 h, 24 h, and 48 h compared to the control. (**A**) 2D-DIGE gel (overlay images). Red circle indicates proteins more abundant at all time-points, orange circle only at 8 h, pink circle indicates proteins more abundant at 24 h and 48 h, blue circle only at 24 h, grey circle only at 48 h, yellow circle at 24 h and 48 h. (**B**) Examples of normalized DIGE spots.

A quantitative DIGE comparison of the above-mentioned proteome profiles led to the detection of 66 differentially abundant protein spots relative to the control (ratio ≥ 2.0, *p* < 0.05 with FDR correction, Figure 2).

**Figure 3:**
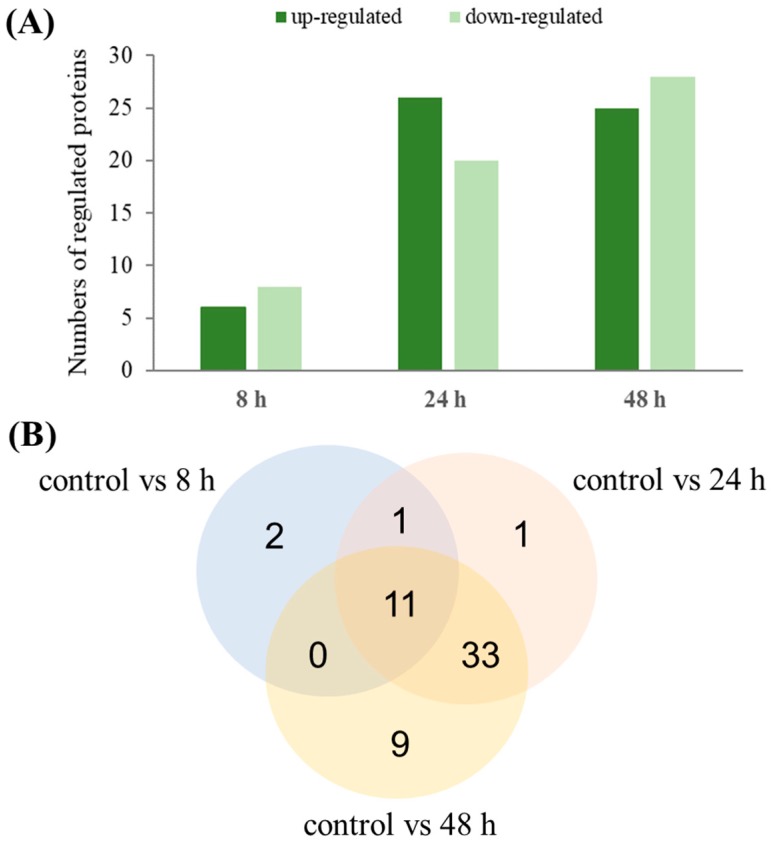
Number of significantly differentially expressed proteins in *Pseudomonas putida* KT2440 during mcl-PHAs synthesis. (**A**) Number of up-regulated and down-regulated genes at 8 h, 24 h, and 48 h of the cultivation compared to the control. (**B**) Venn diagram showing the numbers of common and specific differentially expressed proteins during mcl-PHAs synthesis.

Of the 66 differentially abundant protein spots, 57 were identified using MALDI TOF/TOF analysis (Table S1, supplementary materials). These spots represented 55 proteins, for which two corresponded to two proteoforms, such as isopropylmalate isomerase large subunit (spot 320 and 325) and catalase/peroxidase HPI (spot 210 and 212). As shown in Figure 3A, the number of proteins induced in *P. putida* cells at 8 h, 24 h, and 48 h were 6, 26, and 25, respectively, while the number of repressed genes were 8, 20, and 28, respectively. During all three time-points of the bioprocess 11 proteins showed a changed expression profile. Among them, 5 proteins were induced, 4 proteins were repressed and 2 proteins were up- or down-regulated depending on the analyzed time-point (Figure 3B). Our data indicate that the number of differentially abundance proteins increased at 24 h and 48 h, indicating that the analysis covered a wide range of proteins with abundant expression in response to mcl-PHAs synthesis and nitrogen limitation. 

**Figure 4:**
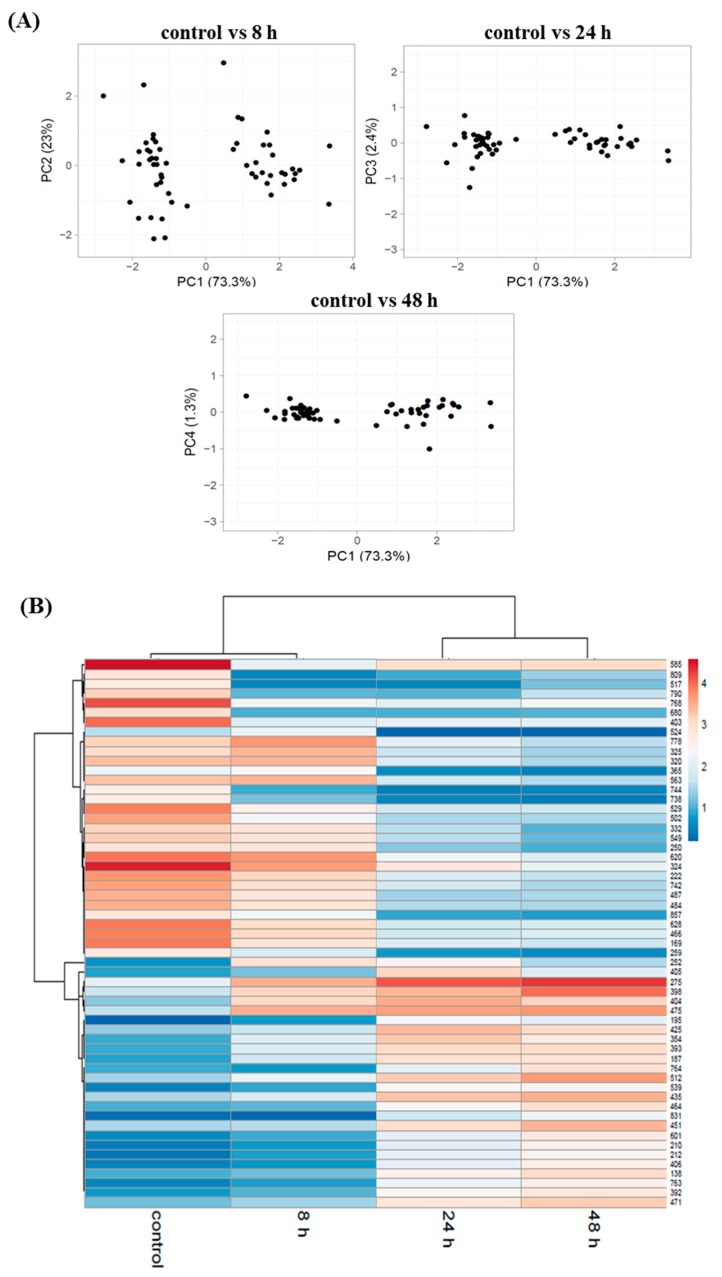
Protein abundance analysis in *Pseudomonas putida* KT2440 during mcl-PHAs synthesis. (**A**) PCA plots of the 2D-DIGE proteomics profile at 8 h, 24 h, and 48 h of the bioprocess compared to the control. (**B**) Cluster analysis of the normalized proteins spots abundance that were differentially regulated at 8 h of the non-limited cultivation (treated as a control) and at 8 h, 24 h, and 48 h of the growth under nitrogen-limited conditions. The cluster analysis resulted in a dendrogram indicated on the left of the heatmap. Every horizontal line indicates one protein spot.

Principal component analysis showed differences in the abundance of the identified proteins spots at 8 h, 24 h, and 48 h compared to the control (Figure 4A). In the PCA plot (Figure 4A) and in the hierarchical cluster analysis in Figure 4B, the sample from 8 h of the bioprocess was closer to the control. Furthermore, the proteins profile from 24 h and 48 h are closer to each other than to the control and to 8 h of the cultivation. It was related with cellular response of Pseudomonas putida KT2440 to nitrogen starvation. At 8 h of the fermentation the nitrogen was still available in the growth medium similarly to the control, whereas at 24 h and 48 h the nitrogen source was depleted.

**Figure 5:**
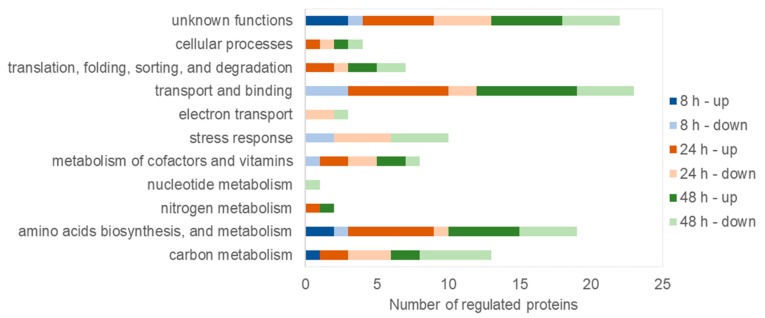
Classification of the identified proteins into role categories.

The proteins that were differentially expressed during mcl-PHA synthesis under nitrogen depletion were categorized into functional classes (Figure 5). The analysis showed that amino acids, biosynthesis, and metabolism, and transport and binding were the top detected functional categories in all analyzed time-points. Moreover, the proteins identified at these time-points were related to carbon metabolism, stress response, and metabolism of cofactors and vitamins. In addition, functional categories included nitrogen metabolism, electron transport, translation, folding, sorting and degradation, and cellular processes were significantly differed at 24 and 48 h of the fermentation. Furthermore, the protein associated with nucleotide metabolism was repressed only at 48 h of the bioprocess. Potential functions of these differentially regulated proteins in response to nitrogen starvation during mcl-PHA synthesis are described below.

### 3.4. Activities of Proteins during Mcl-PHA Synthesis under Nitrogen Deficiency

Many species belonging to the *Pseudomonas* genus have been reported to possess the abilities of synthesizing mcl-PHA from fatty acids. When fatty acids were used as the only substrate, the β-oxidation pathway plays an important role in providing intermediates for cell growth and biopolyesters synthesis [20]. It was proven that the fatty acid β-oxidation cycle is closely related with mcl-PHA synthesis, therefore, many studies have been conducted to metabolically engineer its genes to increase PHA accumulation. In particular, two proteins, 3-ketoacyl-CoA thiolase (FadA) and 3-hydroxyacyl-CoA dehydrogenase (FadB) were taken into account as the important enzymes catalyzing the last two steps in the β-oxidation pathway [21]. Our study revealed that during mcl-PHAs synthesis process these proteins had a significantly altered abundance. As reported from the proteomic analysis, 3-ketoacyl-CoA thiolase (spot 487) and 3-hydroxyacyl-CoA dehydrogenase (spot 222) were significantly downregulated at 24 h and 48 h of the bioprocess relative to the control. The above-mentioned proteins seem to play an important role in controlling the structure of mcl-PHAs [22]. Our results showed that a decrease of 3-HDD molar fraction could be assigned to these proteins. Also, Ouyang et al. [23] observed the changes in the monomer composition when *fadB* and *fadA* genes were knocked out in *P. putida* KT2442.

Isocitrate lyase (spot 365) involved in glyoxylate bypass was hampered when mcl-PHAs content in *P. putida* cells was high and nitrogen was completely knocked out (at 24 h and 48 h). In particular, the abundance of this protein at 48 h decreased 4-fold as compared to that in the control. This observation suggested a change in the carbon flux from glycolytic pathway to tricarboxylic acid (TCA) cycle. At the same time-points, isocitrate dehydrogenase (spot 393) tended to be highly expressed suggesting that there was a need to replenish the cellular NADH pool. Previous studies showed that elevated ratios of NADH/NAD^+^ were essential for PHAs biosynthesis in *Pseudomonas putida* U [24] or *Alcaligenes eutrophus* [25]. 

The mcl-PHA biosynthesis process under stressful conditions requires a high energy demand. Therefore, besides an increased activation of protein involved in the TCA cycle, *P. putida* KT2440 activated the arginine deaminase pathway as an alternative for energy production. During this pathway arginine is converted to ornithine, ammonia and CO_2_ generating 1 mol of ATP per mol of arginine consumed. In our study, the abundance of arginine deiminase was increased during the time-course of the bioprocess. The activation of proteins of the arginine deiminase pathway seems to be a way to produce energy under unfavorable growth conditions [26]. Furthermore, to cope with the higher PHA synthesis demand, the above-mentioned pathway was activated to replenish the pool of energy that was lost to PHA formation. 

Furthermore, during mcl-PHA synthesis under nitrogen starvation, nitrogen regulatory protein P-II was increased in abundance 5.6-fold and 7.2-fold at 24 h and 48 h relative to the control, respectively. It is a small signal transduction protein commonly known as one of the most conserved signal proteins in bacteria. Moreover, this protein family has been found to play an important role in the coordination of nitrogen metabolism by controlling nitrogen-assimilatory processes [27]. It is known to control the activities of a diverse range of enzymes, transcription factors, and membrane transport proteins [28]. The expression of this protein was related with a need to increase a pool of nitrogen from alternative sources due to the nitrogen deficient cultivation conditions that were applied to increase PHA biosynthesis during the growth of *P. putida* KT2440.

Mcl-PHAs biosynthesis process and nitrogen starvation induced the expression of a number of stress-related proteins. Stress conditions may trigger responses that favor PHA synthesis but PHA accumulation confers survival and stress tolerance in a changing environment. The abundance of catalase/peroxidase HPI protein (spot 210, 212) increased during the time-course of the mcl-PHAs synthesis. This enzyme is related with the oxidative defense system of bacterial [29]. Its upregulation could be affected by the oxidative stress during the growth of bacteria due to the imbalance in carbon and nitrogen assimilation and may be expressed for detoxification of reactive oxygen species (ROS) [30]. However, other stress related proteins like anti-oxidant AhpCTSA family protein (spot 680) and OsmC family protein (spot 809) were down-regulated. Moreover, the abundance of heat-shock protein 90 (spot 250), and cold shock protein CspA (spot 857) were reduced at 24 h and 48 h of the cultivation. Furthermore, chaperonin GroEL (spot 324) was down-regulated at the end of the bioprocess. It was shown that these proteins are also needed during non-shock temperature growth. Heat-shock proteins assist the correct protein folding and aggregation, whereas cold shock protein is essential for the maintenance of membrane fluidity and protein synthesis [31]. Czapski and Trun [32] confirmed that in *E. coli*, both growth medium and growth phase have been shown to affect the transcription of *csp* genes. Moreover, the abundance of cold shock protein CspA could be associated with growth phase of bacteria. In our experiment, the *Pseudomonas putida* KT2440 cells were in late logarithmic phase at 24 h of the cultivation, whereas stationary phase was detected at 48 h. Similar observations have been made in *Escherichia coli*, indicating that transcripts of *cspA* predominate during the lag phase and first stages of logarithmic growth, whereas only small levels of *cspA* mRNA can be detected at other stages of growth [33].

Under nitrogen starved conditions together with an increase in mcl-PHAs content in *P. putida* KT2440 cells, the overexpression of elongation factor G (spot 187) was observed. It assists the ribosome in synthesizing proteins participating in the translocation step of the elongation phase and in ribosome disassembly following termination [34]. Also, transcription termination factor Rho was up-regulated at 24 and 48 h compared to the control. Rho-dependent termination is responsible for proper transcript termination, which is essential for accurate gene expression and the removal of RNA polymerase (RNAP) at the ends of transcription units [35]. The synthesis of ribosome protein (50S ribosomal protein L7/L12, spot 742) was down-regulated at 24 h and 48 h of the fermentation relative to the control. This protein is involved in interaction with translation factors during protein biosynthesis being essential for optimal translation rates, accuracy, and termination [36]. 

Our results suggested that nitrogen limitation enhanced ABC transporter activity, which was involved in the absorption of amino acids and polyamine for maintaining the essential cellular functions during the growth under nutrient-deplete conditions. In contrast, proteins responsible for transport of specific compounds like glutamate/aspartate ABC transporter (spot 585) and dipeptide ABC transporter (spot 778) were repressed. Moreover, membrane proteins were differentially expressed during mcl-PHAs biosynthesis process. The abundance of outer membrane ferripyoverdine receptor (spot 169) was decreased at 24 h and 48 h of the bioprocess. Pyoverdine-mediated iron uptake by the FpvA receptor in the outer membrane of *Pseudomonas aeruginosa* was shown to be dependent on the inner membrane protein TonB. Our results indicate that TonB-dependent receptor (spot 138) was up-regulated suggesting that could inhibit the transport of ferric pyoverdine by FpvA. Such interaction of TonB with the outer membrane receptor FpvA of *Pseudomonas aeruginosa* was also observed by Adams et al. [37]. The authors suggested that an interference with endogenous TonB1 was caused by competition for binding sites at the transporter or by formation of nonfunctional TonB heterodimers. Furthermore, the outer membrane porin F (OmpF, spot 451) was overexpressed at 24 h and 48 h suggesting that there was a need to support passive diffusion of small molecules through the outer membrane. Also, the abundance of flagellin FliC decreased significantly by 3.4-fold and 4.5-fold compared to the control at 24 h and 48 h of the bioprocess, respectively. Previous studies showed that it plays an essential role in normal flagellum function, bacterial growth, protein secretion by TTSS, and bacterial virulence [38]. Furthermore, cell division protein FtsZ was significantly up-regulated up to the end of the fermentation. This protein was found to be the first protein to localize to the site of future division in bacteria [39]. Moreover, adenylate kinase (spot 620) was down-regulated at the end of the process. This protein is important for cellular growth and survival, for regulation of adenine nucleotide homeostasis as a major energy source in bacterial cells [40]. This suggests that *Pseudomonas putida* KT2440 slowed down the metabolic processes when the nitrogen source was depleted and mcl-PHAs content was high. 

## 4. Conclusions

In the present study, the proteomic changes associated with nitrogen deprivation as a trigger for enhancing medium-chain-polyhydroxyalkanoate synthesis was addressed in *Pseudomonas putida* KT2440 grown on oleic acid. It can be concluded that nitrogen starvation increased the mcl-PHAs content in bacterial cells. A 2D-DIGE experiment allowed us to identify differentially abundant proteins during mcl-PHAs synthesis. The analyses showed that the proteins involved in carbon metabolism were significantly affected under nitrogen depletion and are essential for PHA synthesis and accumulation. In spite of an increased activation of protein involved in the tricarboxylic acid cycle, *P. putida KT2440* activated the arginine deaminase pathway as an alternative for energy production under stressful conditions. Furthermore, our results confirmed that 3-ketoacyl-CoA thiolase and 3-hydroxyacyl-CoA dehydrogenase could control the monomeric composition of mcl-PHAs. Moreover, during the time-course of the mcl-PHAs synthesis the proteins involved in stress response and cellular homeostasis changed their abundance. Our results also suggested that in order to maintain the crucial cellular functions during growth, *Pseudomonas putida* KT2440 cells enhanced ABC transporter activity to absorb amino acids and polyamine under nutrient-deplete condition. The obtained results improved our knowledge of the molecular mechanisms underlying the induction of mcl-polyhydroxyalkanoates synthesis and accumulation in *Pseudomonas putida* KT2440 that could lead to improved strategies for industrial production of PHAs.

## Supplementary Materials

The following are available online at https://www.mdpi.com/2073-4360/11/5/748/s1, Table S1 Significantly differentially expressed proteins under nitrogen starvation during mcl-PHAs synthesis in *Pseudomonas putida* KT2440 identification by MALDI-TOF/TOF analysis.
