# Supplementary material for: Time-Course Proteomic Analysis of Pseudomonas putida KT2440 during Mcl-Polyhydroxyalkanoate Synthesis under Nitrogen Deficiency
Source: Polymers (Basel). 2019 Apr 26;11(5):748. doi: 10.3390/polym11050748 (PMC6571654; doi:10.3390/polym11050748)
Supplement: Supplementary file 1 [file polymers-11-00748-s001.pdf]

Table S1 Significantly differentially expressed proteins under nitrogen starvation during mcl-PHAs synthesis in *Pseudomonas putida* KT2440 identification by MALDI-TOF/TOF analysis

Spot	Accession	Protein name	pI/Mw(Da)	Mascot score	Coverage (%)	Fold change		
						8 h/ control	24 h/ control	48 h/ control
**Carbon metabolism**								
332	gi|26987633	hydro-lyase. Fe-S type. tartrate /fumarate subfamily. alpha subunit	5.14/55.017	224	15	nd	nd	-2.95
475	gi|26989059	methylcitrate synthase	6.24/42.310	308	27	2.16	2.25	2.31
393	gi|26990716	isocitrate dehydrogenase. NADP-dependent	5.46/45.781	233	25	nd	3.12	3.12
365	gi|26990810	isocitrate lyase	5.38/48.888	239	28	nd	-3.40	-4.09
484	gi|26990459	beta-ketothiolase	6.41/41.133	428	57	nd	-2.27	-2.37
487	gi|26988861	3-ketoacyl-CoA thiolase	6.53/41.814	155	19	nd	-2.38	-2.34
222	gi|26988860	3-hydroxyacyl-CoA dehydrogenase	5.81/77.629	206	40	nd	nd	-2.32
**Nitrogen and amino acids biosynthesis and metabolism**								
404	gi|26987737	arginine deiminase	5.57/46.775	367	49	2.37	2.69	2.43
831	gi|26991910	nitrogen regulatory protein P-II	5.42/12.323	322	62	nd	5.58	7.19
517	gi|26989176	L-asparaginase. type II	6.99/38.584	346	50	-4.76	4.63	-2.20
549	gi|26991362	ketol-acid reductoisomerase	5.48/36.575	396	48	nd	-2.05	-2.85
320	gi|26988711	isopropylmalate isomerase large subunit	5.55/51.679	159	36	nd	nd	-2.25
325	gi|26988711	isopropylmalate isomerase large subunit	5.55/51.679	249	27	nd	nd	-2.14
512	gi|26991343	gamma-glutamyltransferase	5.79/59.854	223	11	nd	2.21	2.50
405	gi|26990739	NADP-dependent dihydropyrimidine dehydrogenase subunit	5.44/49.526	248	33	nd	3.62	2.27
**Nucleotide metabolism**								
620	gi|26988238	adenylate kinase	5.59/23.322	312	44	nd	nd	-2.01
**Metabolism of cofactors and vitamins**								
790	gi|26991799	phosphopantetheine adenylyltransferase	6.98/17.983	218	78	-2.92	-3.01	nd
764	gi|26987223	bacterioferritin	4.80/18.075	164	46	nd	2.23	3.08
138	gi|499254267	TonB-dependent receptor	5.66/10.074	143	21	nd	2.51	3.03
466	gi|519035676	Re/Si-specific NAD(P)(+) transhydrogenase subunit alpha	5.65/39.128	193	36	nd	-2.20	-2.02
**Stress response**								
212	gi|26990379	catalase/peroxidase HPI	5.64/82.068	310	21	2.08	5.34	6.29
210	gi|26990379	catalase/peroxidase HPI	5.64/82.068	200	15	nd	4.31	5.58
680	gi|26987820	anti-oxidant AhpCTSA family protein	5.06/21.887	404	69	-3.03	-3.01	-2.84
809	gi|26988589	OsmC family protein	6.28/14.770	192	43	-4.98	-2.99	-2.06

Spot	Accession	Protein name	pI/Mw(Da)	Mascot score	Coverage (%)	Fold change		
						8 h/ control	24 h/ control	48 h/ control
250	gi|26990871	heat shock protein 90	5.21/71.567	449	41	nd	-2.21	-2.91
324	gi|26988095	chaperonin GroEL	4.97/56.765	168	34	nd	nd	-2.07
857	gi|26989186	cold shock protein CspA	6.56/77.06	171	57	nd	-3.15	-3.35
**Electron transport**								
768	gi|26991550	azurin	6.41/16.189	163	42	nd	-2.09	nd
529	gi|26990893	electron transfer flavoprotein. alpha subunit	5.07/31.218	303	62	nd	-2.17	-2.24
**Transport and binding**								
744	gi|26987920	outer membrane protein H1	6.28/21.545	118	46	-2.72	-5.40	-4.60
403	gi|26987619	outer membrane porin	6.13/50.226	218	16	-2.08	nd	nd
585	gi|26987807	glutamate/aspartate ABC transporter. periplasmic amino acid-binding protein	8.61/33.521	237	50	-2.12	nd	nd
601	gi|26987038	glycine betaine/L-proline ABC transporter. periplasmic binding protein	6.22/34.661	259	32	nd	3.51	4.60
406	gi|26991521	branched-chain amino acid ABC transporter. periplasmic amino acid-binding protein. putative	5.83/46.086	236	17	nd	3.86	4.81
539	gi|26988032	general amino acid ABC transporter. periplasmic binding protein	5.84/36.630	136	36	nd	4.47	4.65
464	gi|26987153	polyamine ABC transporter. periplasmic polyamine-binding protein	5.62/37.891	355	37	nd	2.41	2.96
471	gi|26988458	ABC transporter. periplasmic binding protein	5.81/38.669	209	59	nd	2.36	2.69
778	gi|26987621	dipeptide ABC transporter. periplasmic peptide-binding protein	6.64/60.556	83	15	nd	nd	-2.05
451	gi|26988814	OmpF family protein	4.72/37.217	222	34	nd	2.01	2.30
435	gi|26991547	extracellular ligand-binding receptor	5.93/40.417	324	39	nd	2.22	2.31
169	gi|26990909	outer membrane ferripyoverdine receptor	5.46/89.784	253	41	nd	-2.01	-2.28
563	gi|26991257	AAA family ATPase	5.69/32.183	289	53	nd	nd	-2.15
**Translation, folding. sorting and degradation**								
742	gi|26987187	50S ribosomal protein L7/L12	4.74/12.592	142	19	nd	-2.00	-2.43
187	gi|26990806	elongation factor G	5.25/78.011	242	15	nd	3.35	3.25
398	gi|26991890	transcription termination factor Rho	7.71/47.126	122	37	nd	2.09	2.41
**Cellular processes**								
425	gi|26988076	cell division protein FtsZ	4.98/41.918	460	30	nd	2.49	2.23
259	gi|26991067	flagellin FliC	4.39/67.806	87	7	nd	-3.43	-4.54
**Other functions**								
354	gi|37222601	D-hydantoinase	5.71/52.766	170	25	2.06	3.45	2.84
195	gi|26988393	dehydrogenase subunit. putative	5.54/81.426	150	17	3.57	10.51	9.38
738	gi|26989162	alkyl hydroperoxide reductase. C subunit	4.98/20.608	174	80	-2.20	-4.74	-5.36

Spot	Accession	Protein name	pI/Mw(Da)	Mascot score	Coverage (%)	Fold change		
						**8 h/ control**	**24 h/ control**	**48 h/ control**
252	gi|26987139	putative serine protein kinase. PrkA	5.74/74.022	453	23	4.19	3.58	-2.13
275	gi|26987501	hypothetical protein PP_0765	5.18/68.123	336	18	nd	nd	2.04
524	gi|26990488	syringomycin biosynthesis enzyme 2	5.55/35.030	295	48	nd	-7.73	-7.48
763	gi|26987000	regulator of murein cross-linking	5.43/15.606	425	77	nd	3.94	4.71
392	gi|26988391	hypothetical protein PP_1659	5.71/48.705	216	20	nd	3.11	3.61
628	gi|26990631	short chain dehydrogenase	6.89/26.989	198	40	nd	-2.17	-2.07
502	gi|26988421	aromatic hydrocarbon degradation protein	5.44/45.665	269	269	nd	-2.40	-2.44

nd – not detected as significantly difference
